# Phimosis Presenting With Preputial Calculus: A Case Report on an Uncommon Presentation of a Common Condition

**DOI:** 10.7759/cureus.12475

**Published:** 2021-01-04

**Authors:** Bala Murugan, Snehasis Das, Mohammed Aslam M

**Affiliations:** 1 Department of Surgery, Jawaharlal Institute of Postgraduate Medical Education and Research, Puducherry, IND

**Keywords:** calculi, preputial calculi, phimosis

## Abstract

Preputial calculus is a rare clinical entity in the realm of urolithiasis. It is usually seen in elderly people with poor hygiene.^ ^Experts postulate the formation to be driven by nidus deposition, accumulation of inorganic salts, and transmigration of stones along the urogenital system. An X-ray of the pelvis can confirm the diagnosis and surgical intervention can assuage the symptoms. In this case report, we describe an elderly male who presented with a hard penile swelling with a prior history of phimosis for a decade. He underwent an X-ray of the pelvis which helped diagnose a case of preputial calculus. We performed an emergency dorsal slit for the prompt removal of the calculus with follow up debridement. In conclusion, a clinical rarity such as this needs apt diagnosis and surgical mediation to treat it. Follow-up should be considered in the elderly given the possibility of malignant transformation in the future.

## Introduction

Phimosis is a common urological condition encountered by surgeons. Preputial calculus formation is a rarity on the spectrum of urolithiasis conditions. It is usually seen in elderly males with phimosis or poor hygiene. Stones in the prepuce can occur due to migration of the stone from the proximal urinary tract or they may form de novo [1, 2]. It has been postulated that these calculi originate from inspissated smegma and trapped lime salts in the phimotic prepuce, infected stagnated urine, or calculi migration from the upper urinary tract into the preputial sac [1]. Preputial stone formation due to phimosis is a rare condition with less than 15 case reports in the literature. We report this case to outline the fact that preputial calculus should be suspected in elderly patients presenting with a hard penile swelling and phimosis, or other lower-urinary-tract obstruction symptoms.

## Case presentation

A 55-year-old male with a known case of hypertension under regular treatment presented to the casualty department with complaints of swelling of the penis for the preceding two weeks, burning micturition for the preceding 10 days, and inability to pass urine for the preceding two days. He also had lower abdominal pain for two days which was the colicky type with no history of genital trauma. He had a history of inability to retract prepuce for 10 years with multiple episodes of burning micturition for the last one year. He had no fever spikes, reduced urine output, high colored urine, haematuria, and pyuria. He had complaints of obstructive lower urinary tract symptoms including straining, hesitancy, and post-void dribbling for the past one week. He had no significant history of any previous surgeries in the past. He denied a history of any sexual promiscuity in the past. On examination, he was conscious, oriented, and febrile with no signs of dehydration. His pulse rate was 104 per minute with a blood pressure of 140/90 mm Hg. He had lower abdominal tenderness without a palpable bladder. Local examination revealed phimosis with a stony hard swelling over the glans penis under the prepuce with no visualization of the urethral meatus. Penile and scrotal edema were present with bilateral testes palpable and non-tender. He had enlarged tender bilateral inguinal lymphadenopathy.

**Figure 1:**
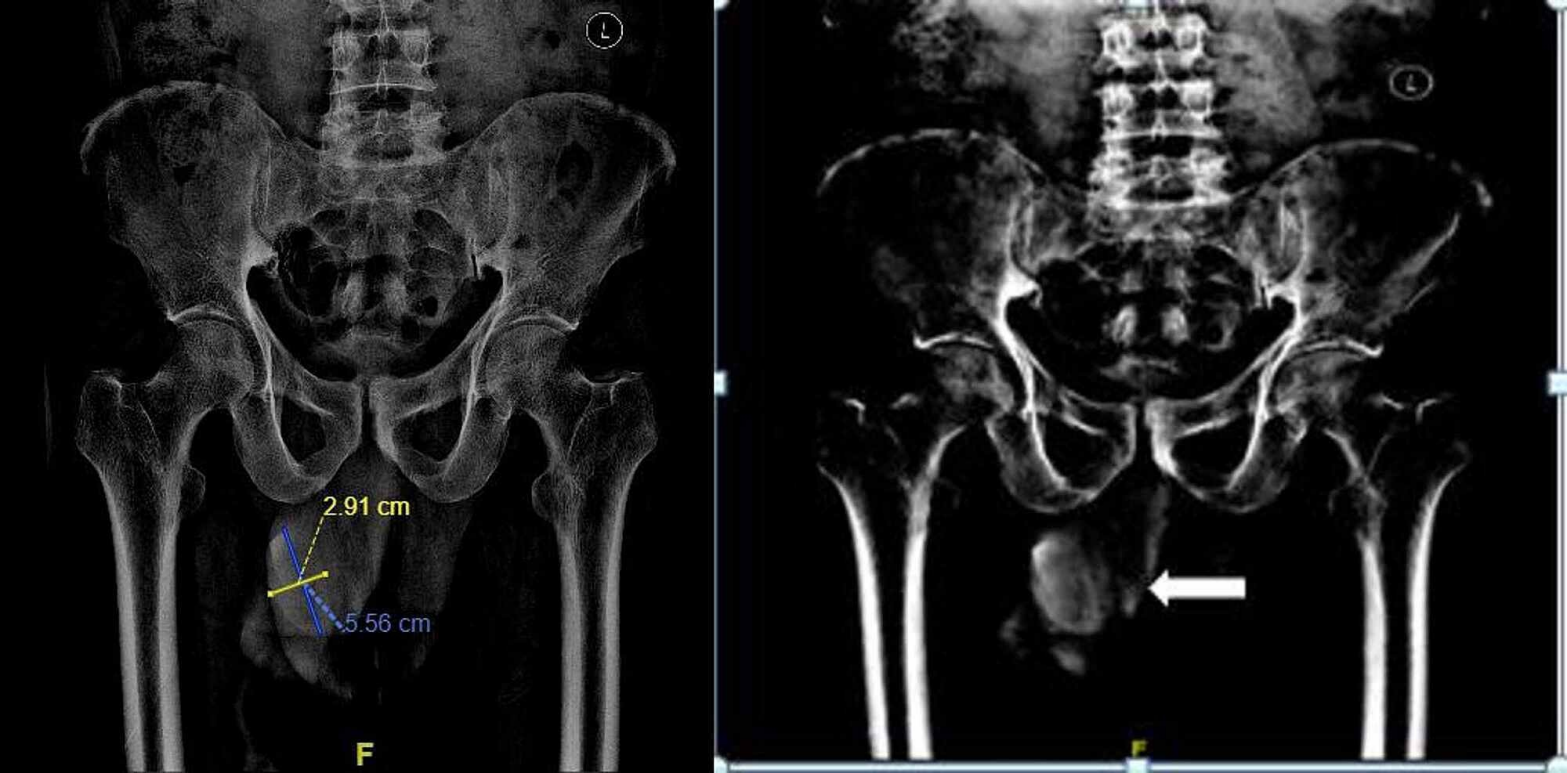
X-Ray pelvis demonstrating the calculus (shown by arrow) over the glans

**Figure 2:**
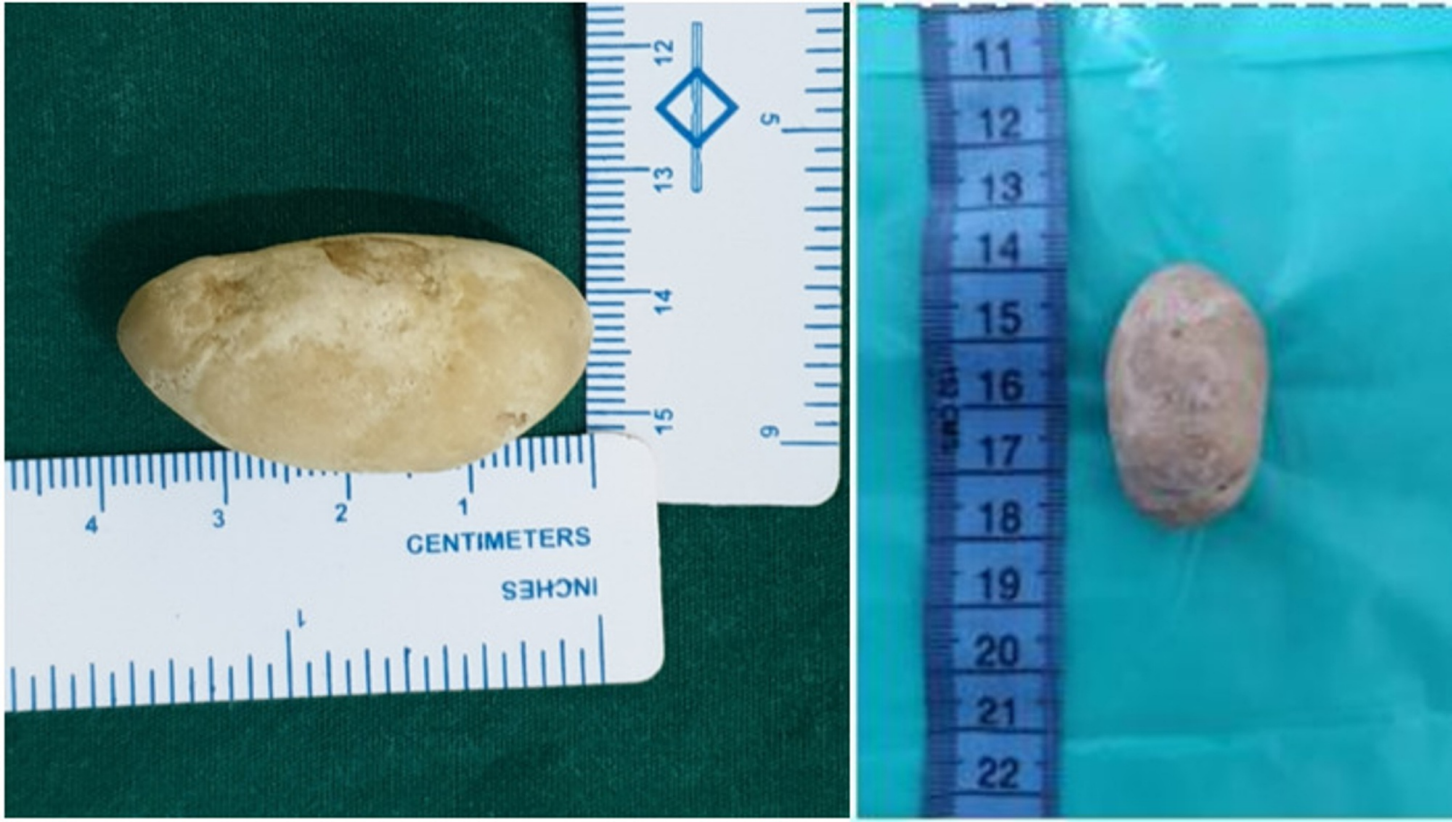
Preputial stone (measuring 4.5 cm x 3 cm)

The initial clinical impression was balanoposthitis with underlying carcinoma of the penis considering his age of presentation. Preputial calculus was also considered as a differential diagnosis in view of the hard consistency of the swelling. X-Ray of the pelvic region showed a radiopaque shadow in the glans region (Figure 1). Ultrasound of the abdomen was done to rule out urolithiasis. Its findings included mucosal irregularity of bladder suggestive of cystitis with no associated renal calculi or hydroureteronephrosis. His creatinine and total white blood cell count were within normal limits and his urine culture was sterile. On the day of admission, cannulation with an infant feeding tube through the meatus was attempted. Finally, a dorsal slit was performed followed by catheterization with a Foley’s 12 Fr catheter. A single stone of size 4.5 cm x 3 cm (Figure 2) was removed from the preputial space. He was started on intravenous ciprofloxacin and metronidazole empirically in view of the clinical diagnosis of balanoposthitis. His wound was debrided and dressed regularly with Eusol. Penile and scrotal swelling gradually subsided with the local application of magnesium sulfate. Foley’s catheter was removed and the patient started passing urine freely from the urethral meatus. The patient was better symptomatically and was discharged on the seventh day.

## Discussion

Preputial calculus is a rare clinical entity in the medical literature. Preputial calculi originate from three probable methods as outlined by Winsbury-White, namely (1) inspissated smegma, (2) combination of smegma and urinary salts, and (3) concentration of urinary salts alone [2]. Wilford has segregated them based on pathogenesis : (1) inspissated smegma with lime salts, (2) struvite composition secondary to infection, and (3) stones formed in the proximal urinary tract which are trapped during migration [3].

Long-standing phimosis with poor local hygiene, underlying neurological or urological anomalies, and geographical distribution (third world countries) are the known risk factors of preputial calculus [1]. Clinical features might vary from strangury, poor flow; prolonged voiding time, haematuria, and associated inguinal lymphadenopathy secondary to infection. Some neglected preputial stones may result in serious complications such as bilateral hydronephrosis and acute renal failure or even penile carcinoma especially in the vulnerable elderly [4]. Furthermore, it leads to urethral fistula and preputial skin fistula [4]. 

Ultrasound or non-contrast computerized tomography of the kidneys, ureters, and bladder is indicated to rule out urolithiasis and ongoing complications like hydroureteronephrosis. Sometimes even a plain pelvic X-Ray shows a radiopaque shadow in the region of glans which aids in the diagnosis as in our case. Treatment is primarily surgical with dorsal slit accompanied with a stone extraction which has been seen to immediately alleviate the obstructive uropathy [3]. In our case, our patient underwent dorsal slit after a failed attempt to cannulate with an infant feeding tube to drain the urine. As a final resort, patients can undergo supra-pubic catheterization if circumcision fails to drain urine. In addition, a stone analysis should be done to delineate any metabolic cause in the patient which would predispose him to the formation of stones in the urogenital system [3].

In our patient, who presented with obstructive uropathy to the casualty department, immediate radiography aided in securing the diagnosis of preputial calculus, and a prompt surgical treatment alleviated the symptoms. A possible mechanism for the formation of the calculus in our patient seems to be the deposition of inspissated smegma and salts into the anatomical nidus. Stone analysis of the calculus revealed a composite of calcium carbonate and calcium sulfate-the most common type [3]. Daily dressing and serial debridement of the wound after stone extraction hastened the patient’s recovery and he was discharged asymptomatic at the end of one week. In the case of deferral of treatment in our patient, given his age, he might have presented later as a case of urogenital carcinoma.

## Conclusions

Preputial calculi are a very rare clinical entity almost invariably occurring in association with phimosis and commonly presenting as a cause of obstructive uropathy with acute retention of urine. Awareness of the condition is essential so that it can be considered with a high degree of suspicion for differential diagnosis in cases of hard penile swelling with associated phimosis. A plain X-ray helps in its diagnosis. A dorsal slit and stone extraction will lead to prompt relief of symptoms. Here, we presented a patient with obstructive uropathy with phimosis and balanoposthitis that was due to a preputial calculus that probably formed due to poor local hygiene and long-standing phimosis. Elderly patients have to be followed up with a high suspicion for malignant transformation for early screening and detection.
